# Traumatic Orbital Emphysema Following Blunt Trauma and Nose Blowing

**DOI:** 10.7759/cureus.48584

**Published:** 2023-11-09

**Authors:** Qi Xiong Ng, Xiao Chien Lim, Jia Cherng Chong, Hanida Hanafi, Lik Thai Lim

**Affiliations:** 1 Ophthalmology, Hospital Tengku Ampuan Rahimah, Klang, MYS; 2 Family and Community Medicine, Kaiteki Skin Aesthetic Clinic, Kuala Lumpur, MYS; 3 Ophthalmology, Hospital Queen Elizabeth, Kota Kinabalu, MYS; 4 Ophthalmology, Universiti Malaysia Sarawak, Kuching, MYS

**Keywords:** lamina papyracea dehiscence, case report, black eyebrow, nose blowing, blunt ocular trauma, orbital emphysema

## Abstract

Orbital emphysema commonly resolves with no morbidity. However, sight-threatening complications, such as central retinal artery occlusion and ischemic optic neuropathy, may occur, which can result in poor visual outcomes. Plain skull X-ray, which is widely available, is a useful tool in identifying orbital emphysema. We report a case of a 29-year-old gentleman with underlying allergic rhinitis who presented with a painless, progressively increasing periorbital swelling of the right eye, which was aggravated by nose blowing. He had a history of blunt trauma one day prior to the presentation. Visual acuity was unaffected and optic nerve function tests were unremarkable. There was right upper lid swelling with crepitations, right hypoglobus with restricted upward gaze movement, and right conjunctival injection. Intraocular pressure was within normal limits. The posterior segment examination was unremarkable. A plain skull radiograph revealed a “black eyebrow sign” over the right orbit with no obvious orbital wall fracture. Computed tomography of the orbit showed focal indentation over the right lamina papyracea with superior orbito-palpebral emphysema. Systemic antibiotics, steroid nasal spray, and oral antihistamines were initiated with the prohibition of nose blowing. On post-trauma day five, he made an uneventful recovery. High clinical suspicion and thorough clinical examination with the aid of a plain skull radiograph can diagnose orbital emphysema in order for prompt referral to be undertaken to prevent morbidity. Clinicians should consider orbital emphysema as a differential diagnosis for periorbital swelling, especially if there was a preceding trauma.

## Introduction

Orbital emphysema is benign and commonly resolves with no morbidity [1,2]. However, in certain cases where the optic nerve is involved or central retinal artery occlusion occurs due to high intraorbital pressure, ocular morbidity is significant and the visual outcome may be poor. Hence, it is important to treat orbital emphysema as a potential ophthalmic emergency where timely referral to the ophthalmology team is paramount. We report an unusual case of right orbital emphysema in the absence of orbital wall fracture with good resolution and visual outcome.

## Case presentation

A 29-year-old man presented with a one-day history of progressively worsening painless swelling over the right eye after forceful blowing of his nose. There was a history of blunt force trauma a day prior to the presentation where his right eye was accidentally hit by his wife’s elbow. He had a history suggestive of allergic rhinitis since young but has yet to seek medical treatment. He denied any blurring of vision, floaters, or flashes of light. He went to a primary care facility early the next day but was discharged home with reassurance. However, the swelling progressively increased in size hence he went to another primary care facility where a plain skull radiograph was done but showed no evidence of fracture. He was then referred to the ophthalmology team in a tertiary care facility.

**Figure 1:**
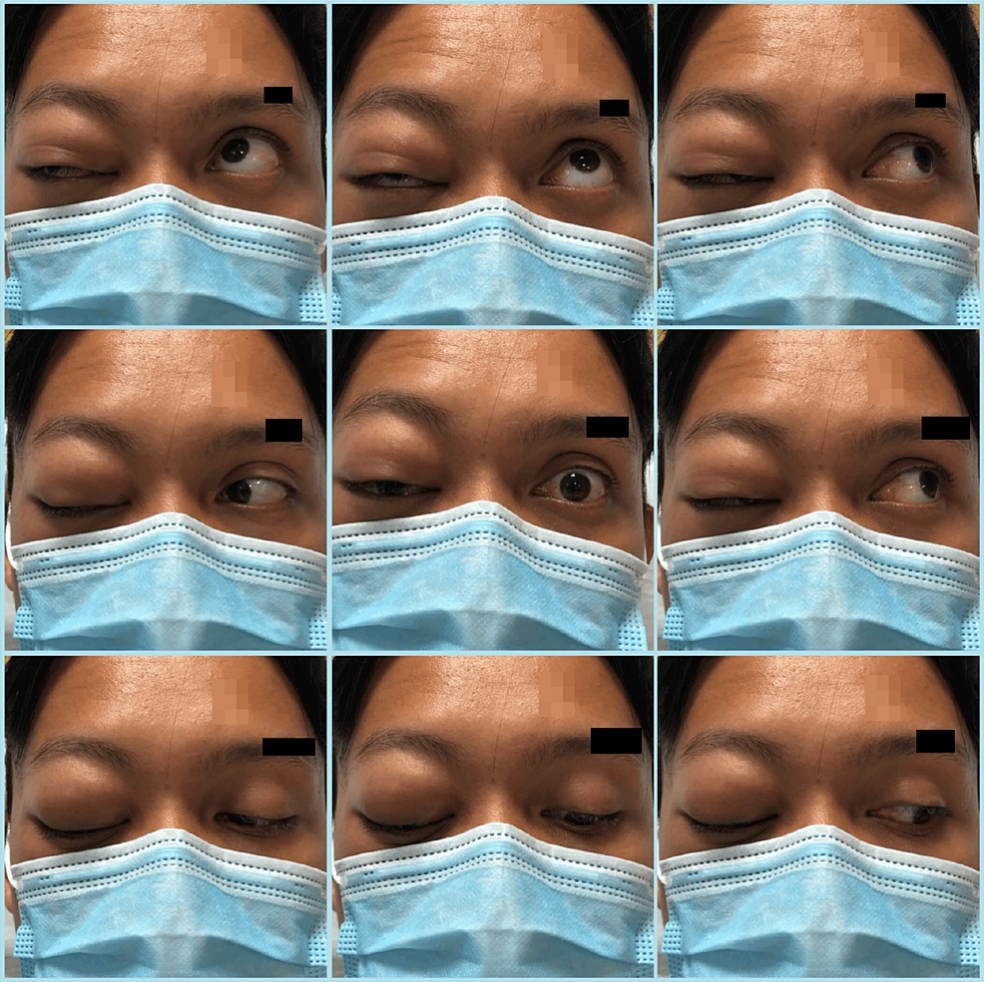
Nine-gaze photographs on the day of presentation showed right periorbital swelling with limitation of elevation over the right eye.

On examination, the patient was alert and oriented. Visual acuity was 6/6 OD (right eye) and 6/9 OS (left eye) using the near vision chart. There was no relative afferent pupillary defect. Upon further examination of the right eye, there was a periorbital swelling with erythematous eyelids, as depicted in Figure 1. On palpation, it was non-tender with subcutaneous crepitation around the swelling. Extraocular muscle movement examination revealed a limitation of elevation over the right eye with no binocular diplopia; the left eye was normal. Right hypoglobus was present. Conjunctiva was injected, the cornea was clear, and the pupil was round, 3 mm, and reactive to light. Intraocular pressure (IOP) was 13 mmHg and 12 mmHg over the right and left eye, respectively. Dilated fundus examination was normal. Anterior and posterior segment examination of the left eye was unremarkable. An otorhinolaryngology consult was made and the team performed a nasoendoscopy, which revealed bilateral inferior turbinate hypertrophy suggestive of allergic rhinitis, otherwise, no other abnormalities were detected.

**Figure 2:**
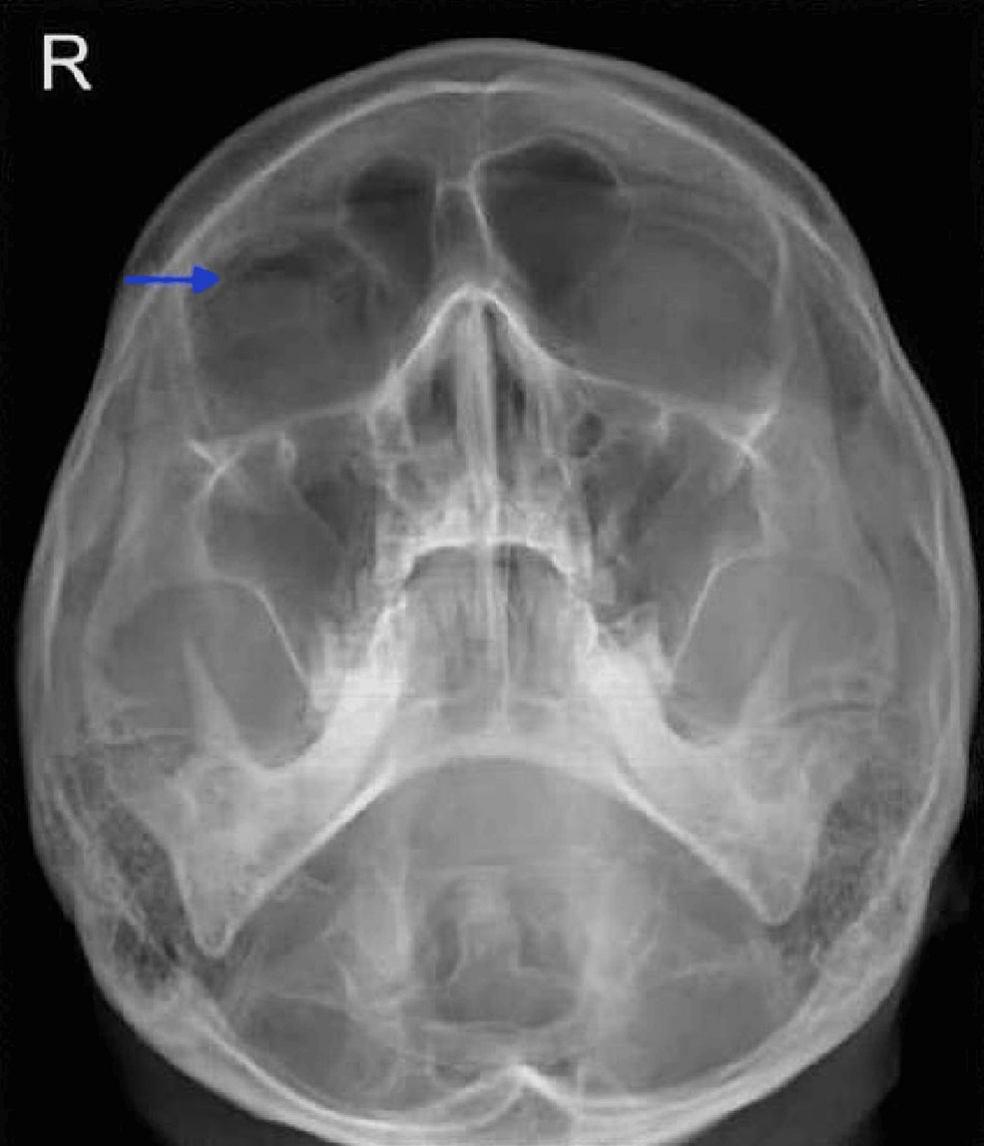
Plain skull X-ray occipitomental view (Waters’ view) showing a “black eyebrow” sign, as depicted by the blue arrow.

**Figure 3:**
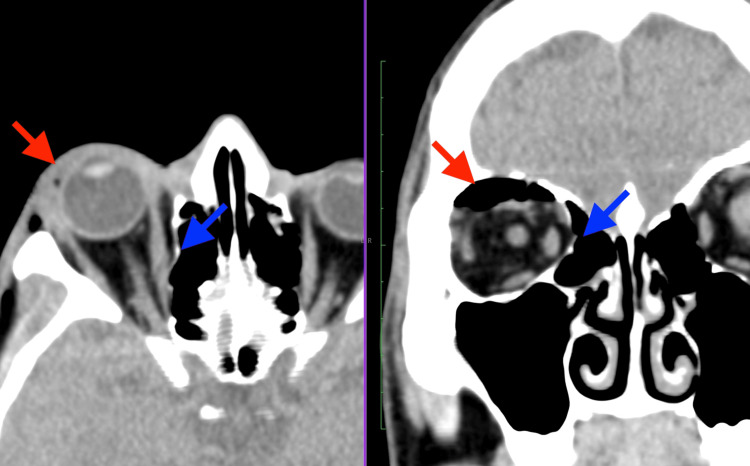
Axial and coronal view of CT soft tissue window show focal indentation of the right lamina papyracea (blue arrows) into the ethmoid sinus and right orbito-palpebral emphysema (red arrows) with air mass at the right superior orbit.

**Figure 4:**
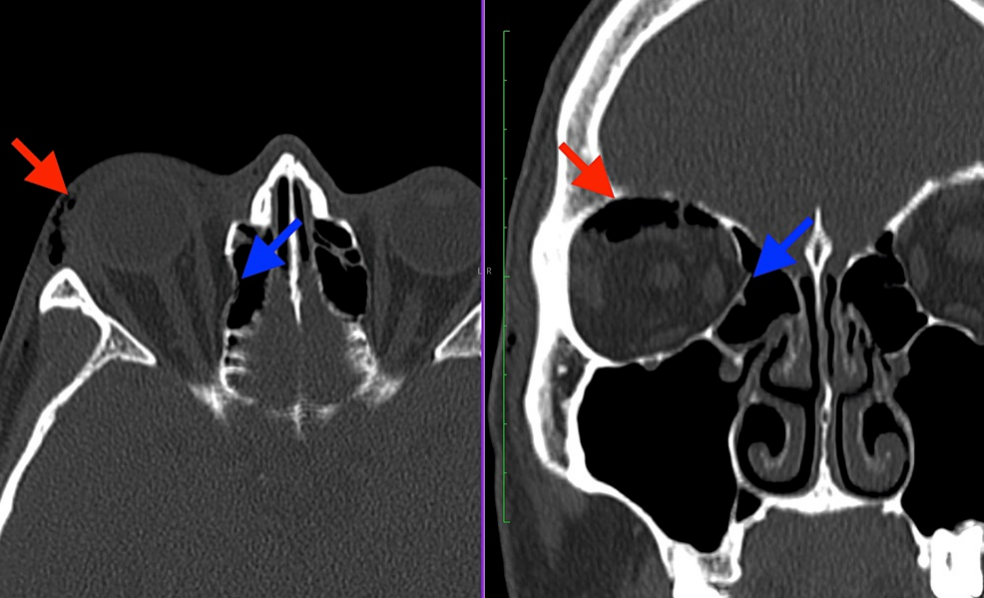
Axial and coronal view of the CT bone window showed focal indentation of the right lamina papyracea (blue arrows) and right orbito-palpebral emphysema (red arrows) similar to the tissue window but showed neither gap at the wall of lamina papyracea nor orbital tissue prolapse, which is suggestive of dehiscence as opposed to fracture.

The plain skull X-ray (Figure 2) from the primary care facility showed the presence of a “black eyebrow” sign [3]. Computed tomography (CT) of the brain and orbit (Figures 3, 4) revealed no obvious wall defect. A focal internal indentation was seen at the medial aspect of the right lamina papyracea into the ethmoid sinus and orbito-palpebral emphysema with a collection of air mass at the right superior orbital space, which is suggestive of dehiscence of the lamina papyracea.

Diagnosis of right orbital emphysema was made and the patient was started on oral cefuroxime 500 mg twice daily for one week and advised on strictly no nose-blowing at home. Steroid nasal spray and oral antihistamines were given.

**Figure 5:**
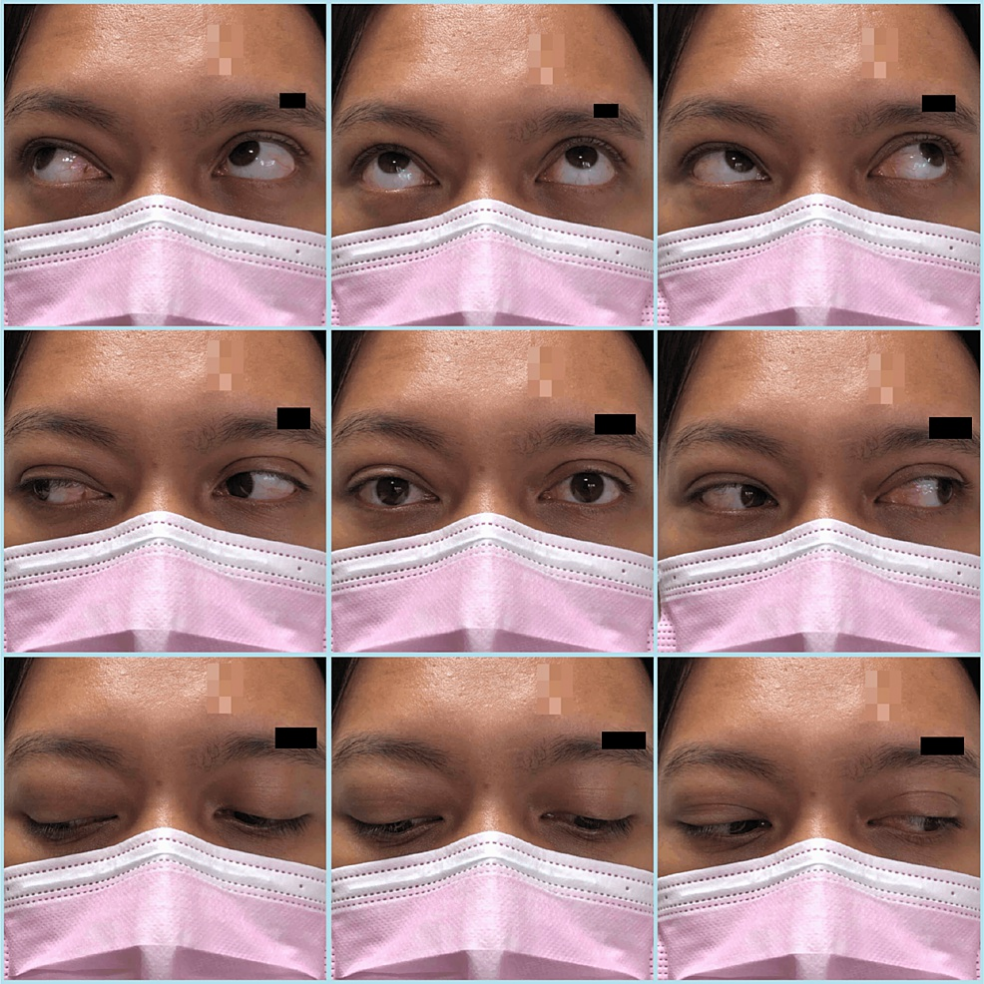
Nine-gaze photographs on post-trauma day five showed resolution of right periorbital swelling with full extraocular muscle motility.

On post-trauma day five, the patient came for review at the ophthalmology clinic and noted that the right periorbital swelling had resolved, as shown in Figure 5. Visual acuity assessment with Snellen chart was 6/6 OD and 6/6 OS. There was no relative afferent pupillary defect. Minimal eyelid swelling was present over the right upper lid with no subcutaneous crepitation. Anterior and posterior segment examinations were unremarkable with IOP of 12 mmHg over both eyes using Goldmann tonometry applanation. Hess chart was performed and there was no evidence of limitation of eye movement.

## Discussion

Orbital emphysema can have variable presentations and can mimic other conditions such as preseptal or orbital cellulitis. Most patients make an uneventful recovery without the need for surgical intervention [2]. In the precedence of trauma and the rapid appearance of worsening periorbital swelling when blowing the nose or sneezing, it may indicate possible communication between the orbit and the nasal cavity or sinus, allowing air to enter the orbit. The orbital tissue, which then occludes the connection, creates a one-way valve effect resulting in the accumulation of air in the orbit [4]. Orbital emphysema is more frequently associated with medial wall fracture [5]. Lamina papyracea dehiscence is an uncommon cause of orbital emphysema. Orbital emphysema may infrequently occur in orbital wall dehiscence with CT evidence of bony defect and is usually precipitated by trauma, nose blowing, or sinusitis [6,7]. The proposed pathophysiology could be due to the difference in the volume of the ethmoid and maxillary sinus or possibly attributable to the difference in length and diameter of the sinusal infundibulii, which obeys Poiseuille’s law of flux resistance [5]. As illustrated by the case in this article, the lamina papyracea dehiscence is likely the cause of orbito-palpebral emphysema despite the absence of CT evidence showing direct communication between the orbit and sinus. A detailed examination is warranted and subcutaneous crepitation upon palpation would suggest the presence of palpebral emphysema, which may indicate a breach of orbital septum where the intraorbital air extends anteriorly to the eyelid [1]. An experimental study by Heerfordt found that by injecting air into a cadaver, the mean intraorbital pressure of 40 to 50 mmHg is required to rupture the orbital septum, where the resistance of the septum is the strongest in younger individuals [4,8]. The presence of orbito-palpebral emphysema would rule out acute orbital compartment syndrome [9], which may result in complications such as raised IOP, central retinal artery occlusion (CRAO), and ischemic optic neuropathy.

As free air in the orbit tends to accumulate superiorly [2,10], a plain radiograph, which is easily accessible at the primary care setting, may reveal a “black eyebrow” sign, characterized by crescent-shaped radiolucency superiorly in the orbit, which is highly suggestive of orbital emphysema [3]. This would enable a timely diagnosis and prompt referral to be made to the ophthalmology unit. While reviewing the CT of the orbit, it is important to look for the causes of the communication, which could be a fracture or dehiscence of the orbital wall. Air in the orbital area, seen as low attenuation (dark black) in the CT scan would suggest the presence of communication between the sinuses and the orbit. As the lamina papyracea is the thinnest orbital wall, dehiscence may be present and could well be an anatomical variant. The key radiological features in a CT scan to determine an orbital wall fracture rather than a dehiscence would be the gap in the bone wall and fat in the bulla cells [11].

Orbital emphysema can be classified and managed as described by Roelofs et al. [2] and Hunts et al. [4], as shown in Tables 1, 2, respectively.

**Table 1:** Classification and management of orbital emphysema. IOP: intraocular pressure. Modified from Roelofs et al. [2].

Stages	Features	Management
Mild	No sign of optic nerve compression and visual compromise	Conservative management
Moderate	Early signs of optic nerve compression/mild visual compromise, elevated IOP, moderate proptosis	Consider needle decompression +/- lateral canthotomy, cantholysis
Severe	Signs of marked optic nerve compression/significant visual compromise, elevated IOP, significant proptosis	Consider emergent orbital decompression

**Table 2:** Classification and management of orbital emphysema. IOP: intraocular pressure; CT: computed tomography; IVMP: intravenous methylprednisolone; CRAO: central retinal artery occlusion. Modified from Hunts et al. [4].

Stages	Proptosis/dystopia	Loss of vision	Increased IOP	CRAO	Management	
I	Absent	Absent	Absent	Absent	Prophylactic antibiotics, decongestants, and emphatic instructions against nose blowing	
II	Present	Absent	Absent	Absent	Orbital CT to rule out other intraorbital processes + prophylactic antibiotics, decongestants, and emphatic instructions against nose blowing +/- needle-coupled syringe decompression	
III	Present	Present	Present/absent	Absent	Orbital CT to localize air mass + immediate needle-coupled syringe decompression + IVMP (30 mg/kg load), followed by 15 mg/kg every 6 hours (unless contraindicated)	
IV	Present	Present	Present	Present	Rapid orbital decompression with lateral canthotomy/cantholysis + emergent orbital CT to localize air mass + immediate needle-coupled syringe decompression + IVMP (30 mg/kg load), followed by 15mg/kg every 6 hours (unless contraindicated)	

The majority of cases of orbital emphysema are self-limiting and typically resolve within seven to 10 days [2]. Sight-threatening orbital emphysema such as in stage II-IV or moderate to severe cases requires immediate decompression either through needle-coupled syringe decompression, lateral canthotomy, and cantholysis or orbitotomy. The needle decompression technique is a simple, rapid, and low-risk procedure first described by Linberg, which is later modified by the addition of normal saline into the syringe with the plunger removed, which allows monitoring the release of the air bubbles and prevents inadvertent injury to structures in the orbit due to suction of the needle tip [4].

Prophylactic antibiotic treatment is recommended to prevent orbital cellulitis, which is an uncommon sight and life-threatening complication of orbital wall fracture [4,12]. As the most common etiologic factor of orbital infection is sinusitis, orbital wall fracture with concomitant sinusitis may enhance the development of orbital infection [13]. However, recent retrospective cohort studies revealed patients with orbital wall fractures not given prophylactic antibiotics did not develop a documented orbital infection. Hence, the authors concluded no evidence of antibiotic utilization and suggested withholding antibiotics for low-risk groups, such as those without upper respiratory tract infections and no steroid usage [14,15]. In addition to judicious usage of prophylactic systemic antibiotics, it is imperative for thorough sinusitis and allergic rhinitis assessment as they may increase the risk of orbital infection and predispose to orbital emphysema, respectively.

## Conclusions

This case highlights the importance of high clinical suspicion and utilization of plain skull radiographs to diagnose orbital emphysema in order for prompt management to be undertaken to prevent morbidity. Computed tomography of the orbit should be performed to look for evidence of fractures, and dehiscence and localize the area of air mass in orbital emphysema. Orbital emphysema should always be considered as a differential diagnosis for periorbital swelling, especially if there is a prior history of trauma. Treatment should be tailored according to the stages of the disease to ensure timely and optimal care can be provided to these patients.
